# Association of hepatic steatosis derived from ultrasound and quantitative MRI with prediabetes in the general population

**DOI:** 10.1038/s41598-021-92681-3

**Published:** 2021-06-24

**Authors:** Muhammad Naeem, Robin Bülow, Sabine Schipf, Nicole Werner, Marcus Dörr, Markus M. Lerch, Jens-Peter Kühn, Wolfgang Rathmann, Matthias Nauck, Marcello Ricardo Paulista Markus, Till Ittermann, Henry Völzke

**Affiliations:** 1 Institute for Community Medicine, University Medicine Greifswald, Walther Rathenau Str. 48, 17475 Greifswald, Germany; 2 Department of Internal Medicine B—Cardiology, Intensive Care, Pulmonary Medicine and Infectious Diseases, University Medicine Greifswald, Greifswald, Germany; 3 Department of Gastroenterology, University Medicine Greifswald, Greifswald, Germany; 4 Institute of Diagnostic Radiology and Neuroradiology, University Medicine Greifswald, Greifswald, Germany; 5 Institute for Clinical Chemistry and Laboratory Medicine, University Medicine Greifswald, Greifswald, Germany; 6 DZHK (German Center for Cardiovascular Research), partner site Greifswald, Germany; 7 DZD (German Center for Diabetes Research), Dresden, Germany; 8 Institute and Policlinic for Diagnostic and Interventional Radiology, University Hospital, Carl Gustav Carus University, TU Dresden, Dresden, Germany; 9 Institute for Biometrics and Epidemiology, German Diabetes Center (DDZ), Leibniz Center for Diabetes Research At Heinrich Heine University Düsseldorf, Düsseldorf, Germany; 10 Department of Zoology, University of Malakand, Chakdara, 18800 Pakistan

**Keywords:** Epidemiology, Outcomes research, Biomarkers, Predictive markers, Magnetic resonance imaging

## Abstract

The aim of our study was to investigate the association of hepatic steatosis derived from quantitative ultrasound and magnetic resonance imaging (MRI) with prediabetes in a large population-based study conducted in Northeast Germany. Hepatic steatosis was assessed through transabdominal ultrasound and quantitative MRI. For analysis we included 1622 subjects with MRI who participated in an oral glucose tolerance test and reported no known type 2 diabetes mellitus (T2DM). We classified participants as proposed by the American Diabetes Association: isolated impaired fasting glucose (i-IFG), isolated impaired glucose tolerance (i-IGT), combined IFG and IGT (IFG + IGT), and undiagnosed T2DM. Regression models were adjusted for age, sex body mass index and alcohol consumption. We observed positive associations of hepatic steatosis with glycated hemoglobin, fasting glucose and insulin, 2-h glucose and insulin, as well as homeostasis model assessment-insulin resistance index. Similarly, individuals having hepatic steatosis as defined by MRI had a higher relative risk ratio (RR) to be in the prediabetes groups i-IFG (RR = 1.6; 95% confidence interval (CI) 1.2; 2.2), i-IGT (RR = 3.3, 95% CI 2.0; 5.6) and IFG + IGT (RR = 2.5, 95% CI 1.6; 3.9) or to have undiagnosed T2DM (RR = 4.8, 95% CI 2.6; 9.0). All associations were attenuated when defining hepatic steatosis by ultrasound. Hepatic steatosis is associated with prediabetes and undiagnosed T2DM in the general population. Quantitative liver MRI revealed stronger associations with prediabetes and undiagnosed T2DM compared to ultrasound, which indicates the higher sensitivity and specificity of MRI to determine hepatic steatosis.

## Introduction

Hepatic steatosis is defined as an excessive fat deposition (> 5%) in the liver in the absence of competing liver disease or hepatocellular carcinoma^1^. Hepatic steatosis is highly prevalent affecting 25% of the world population^2^ and up to 70% of patients with type 2 diabetes mellitus^3,4^. The prevalence of ultrasound-determined hepatic steatosis is highest in the Middle East (32%) and South America (30%), lower in Europe (24%), and lowest in Africa (13%)^2^.

Hepatic steatosis occurs usually when lipid storage is increased through hepatic uptake and de novo lipogenesis through fatty acid oxidation and export of lipid in very low density lipoprotein^5^. Hepatic steatosis is strongly associated with insulin resistance^6^ and postprandial hyperinsulinemia indicating its possible role in the pathogenesis of type 2 diabetes mellitus^7^. Furthermore, the association between hepatic steatosis and type 2 diabetes mellitus may be bidirectional as suggested from some studies^8–10^.

Population-based studies defining hepatic steatosis by computed tomography showed significant associations with type 2 diabetes mellitus^8,11^. Likewise, several previous studies demonstrated associations between sonographically determined hepatic steatosis and type 2 diabetes mellitus^9,12–17^. Although being easy to use and non-radiation-based and therefore a suitable method for population-based research, ultrasound has a low sensitivity for detecting mild steatosis, and limitations in the examination of obese individuals^18,19^.

While there is strong evidence that hepatic steatosis is associated with type 2 diabetes mellitus, data regarding the association between hepatic steatosis and prediabetes is inconsistent. Previous cohort studies demonstrated associations between hepatic steatosis defined by ultrasound and prediabetes defined by fasting glucose and 2-h glucose or glycated hemoglobin (HbA1c)^20–23^. One cross-sectional study found an association between hepatic steatosis defined by fatty liver index and prediabetes categories according to American Diabetes Association (ADA) criteria^24^, whereas others did not^16,21^.

To the best of our knowledge there is no population-based study, which investigated the association of hepatic steatosis as defined by quantitative magnetic resonance imaging (MRI) with prediabetes and undiagnosed type 2 diabetes mellitus. From MRI, the proton density fat fraction (PDFF) can be calculated, which is a quantitative marker for liver fat, more accurate than similar markers taken from ultrasound or computed tomography^25^. In addition, MRI is able to differentiate between liver fat and iron^26^ as well as between focal, regional and general steatosis in a single procedure.

Against this background, the aim of our study is to clarify the association of hepatic steatosis assessed through ultrasound and MRI with prediabetes and undiagnosed type 2 diabetes mellitus defined the by oral glucose tolerance test (OGTT) in a large population-based sample.

## Materials and methods

### Study sample

The Study of Health in Pomerania (SHIP) is a population-based project conducted in Northeast Germany. It consists of the two independent cohorts SHIP and SHIP-Trend. For the present study we used baseline data from the second cohort (SHIP-Trend-0). A stratified random sample of 8826 adults aged between 20 and 79 years was drawn, of which 4420 subjects participated between 2008 and 2012 (response 50.1%). Random sample selection into age and sex-strata was facilitated by centralization of local population registries in the German Federal State of Mecklenburg/West Pomerania^27^.

All participants gave written informed consent. The study conformed to the ethical guidelines of the Declaration of Helsinki as reflected in a priori approval by the local ethics committee of the University of Greifswald.

We excluded individuals without MRI examination (n = 2130), those who reported known liver cirrhosis or hepatitis (n = 46), known type 2 diabetes mellitus (n = 461), and participants with missing data in any of the considered variables (n = 37). The final study population consisted of 1746 (913 women) subjects aged 21 to 82 years. From the analysis regarding prediabetes we further excluded all individuals without OGTT (n = 124) resulting in data from 1,622 (840 women) available for analysis of prediabetes.

### General measurements

Sociodemographic characteristics and medical histories were assessed by computer-assisted face-to-face interviews. Height and weight were measured for calculating the body mass index (BMI = weight [kg]/height^2^ [m^2^]). Alcohol intake was evaluated as beverage-specific alcohol consumption (beer, wine, distilled spirits) on the last weekend and last weekday preceding the examination. The mean daily alcohol consumption was calculated using beverage-specific pure ethanol volume proportions^28^.

### Ultrasound

Transabdominal ultrasound of the liver was performed by examiners using a transportable B-mode ultrasound device (vivid I; GE-Healthcare, Waukesha, WI, USA) with a 2.5 MHz ultrasonic transducer. The examiners used a 2-point scale to assess the presence of hepatic steatosis: (0) no steatosis and (1) steatosis. Hepatic steatosis was defined as a hyperechogenic liver pattern in comparison to the renal cortex^27^.

### Magnetic resonance imaging (MRI)

MRI was performed by using a 1.5-Tesla MRI system (Magnetom Avanto, software version VB15; Siemens Healthineers Erlangen, Germany) with a 12-channel phased-array surface coil^29^. Three-dimensional chemical shift encoded gradient-echo data with three echoes and flyback readout gradient were acquired from an axial slab during a single 19-s breath hold. Imaging parameters included repetition time, 11 ms; echo times, 2.4, 4.8, and 9.6 ms; flip angle, 10°; number of signals acquired, one; bandwidth, ± 1065 Hz per pixel; matrix, 224 × 168 × 64; field of view, 410 × 308 mm; parallel imaging effective acceleration factor, 1.8; and section thickness, 3.0 mm.

Offline reconstructions of a PDFF map (including correction for T1 bias and T2* decay) and a transverse relaxation rate (R2*) map (based on T2* decay measurement of PDFF) were performed. Fat and water ambiguities were resolved by using the phase of the acquired data^30^. Parametric maps of PDFF were used for further analyses.

One trained radiologist reviewed the PDFF. Mean fat fraction values were determined at operator-defined regions of interest placed at the center of the liver by using Osirix (v3.8.1; Pixmec Sarl, Bernex, Switzerland). Regions of interest were placed carefully to avoid blood vessels and regions that were obviously contaminated by partial volume effects and motion artifacts^29^. Hepatic steatosis was defined as PDFF > 5%^30^.

### Laboratory measurements

We requested the participants not to eat, smoke or consume caffeine-containing drinks and to avoid sports for ≥ 8 h before the examination, which was completed during the morning hours. Blood was collected by a trained examiner following a standardized protocol, refrigerated to 4–8 °C and shipped on refrigerant packaging within 4 to a maximum of 6 h to the laboratory. Measurements of fasting glucose and 2-h glucose were based on plasma samples^31^. All assays were performed according to the manufacturers’ recommendations by skilled technical personnel. The study laboratory participated in official quarterly German external proficiency testing programs^32^.

Fasting glucose and 2-h glucose levels were measured using a hexokinase method (Dimension Vista, Siemens Healthcare Diagnostics, Eschborn, Germany)^31^. HbA1c was determined by high-performance liquid chromatography (Diamat, Bio-Rad Laboratories, Munich, Germany). Insulin serum values were measured by a chemiluminescence immunoassay (Immulite 2000 Xpi, Siemens Healthcare Diagnostics, Eschborn, Germany). Fasting insulin and 2-h insulin are expressed as µU/ml. The homeostasis model assessment-insulin resistance index (HOMA-IR) was calculated as (fasting insulin [μU/ml] x fasting glucose [mmol/l] / 22.5)^33^. Serum alanine aminotransferase (ALT), aspartate aminotransferase (AST), and γ-glutamyl transpeptidase (GGT) concentrations were measured photometrically (Hitachi 704; Roche, Mannheim, Germany). ALT, AST, and GGT concentrations are expressed as µkatal/l.

### Ascertainment of diabetes and prediabetes

Participants were classified as having type 2 diabetes mellitus if they reported physician’s diagnosis of type 2 diabetes mellitus in the interview or took glucose-lowering medication (Anatomical Therapeutic Chemical (ATC) classification system code A10). For the OGTT, fasting glucose was sampled, and 75 g of anhydrous glucose (Dextro OGT, Boehringer Mannheim, Mannheim, Germany) was given to the participants without diabetes and glucose-lowering agents. Following the criteria of the ADA^34^, we classified individuals as having normal glucose tolerance when they had fasting glucose values < 5.6 mmol/l and 2-h glucose < 7.8 mmol/l. We classified participants as having prediabetes if fasting glucose values were between 5.6 and 6.9 mmol/l (impaired fasting glucose: IFG) and/or 2-h glucose values were between 7.8 and 11.0 mmol/l (impaired glucose tolerance: IGT). We defined three groups of prediabetes: isolated impaired fasting glucose (i-IFG), isolated impaired glucose tolerance (i-IGT), and combined IFG and IGT (IFG + IGT). Undiagnosed type 2 diabetes mellitus was defined as fasting glucose values ≥ 7.0 mmol/l or 2-h glucose ≥ 11.1 mmol/l^31,33^.

### Statistical analysis

Continuous data are reported as median (with 25th and 75th percentiles) and categorical variables as absolute numbers and percentages. Difference between the subjects with and without hepatic steatosis were tested by Wilcoxon rank-sum test for continous data and chi-square test for categorical data. For analyzing the association between hepatic steatosis and continuous markers of glucose metabolism linear regression models were used by calculating β coefficients and 95% confidence intervals (95% CI). For investigating the association between hepatic steatosis and prediabetes groups, multinomial logistic regression was run by calculating relative risk ratios and 95% CI. All models were adjusted for age, sex BMI and alcohol consumption. A value of p < 0.05 was considered statistically significant in all calculations. All statistical analyses were performed by Stata 14.1 (Stata Corporation, College Station, TX, USA).

## Results

Among the study population consisting of 1,746 individuals (913 women) aged 21 to 80 years the prevalence of hepatic steatosis was 37% (95% CI 34%; 39%) by using MRI and 36% (95% CI 33%; 38%) by using ultrasound. Four-hundred-sixty-seven individuals (73%) with hepatic steatosis identified by ultrasound also had hepatic steatosis derived from MRI (Table 1).

**Table 1:** Characteristics of the study population stratified by hepatic steatosis (MRI).

Variables	Number of individuals	Hepatic steatosis derived from MRI		P value
		No (n = 1,106)	Yes (n = 640)	
Age (years)	1746	46 (37; 57)	55 (47, 64)	< 0.001
Male	833	459 (42%)	374 (58%)	< 0.001
Female	913	647 (58%)	266 (42%)	< 0.001
BMI (kg/m^2^)	1746	25 (23; 28)	30 (27; 32)	< 0.001
Alcohol consumption (g/day)	1732	4 (1; 9)	5 (1; 14)	< 0.001
**Hepatic steatosis ultrasound**				< 0.001
Negative	1119	949 (86%)	170 (27%)	
Positive	619	152 (14%)	467 (73%)	
Liver fat (MRI %)	1746	2.6 (2; 3.5)	9.4 (6.7; 15.2)	< 0.001
ALT (µkatal/l)	1745	0.33 (0.25; 0.43)	0.49 (0.36; 0.67)	< 0.001
AST (µkatal/l)	1743	0.27 (0.21; 0.33)	0.32 (0.26; 0.40)	< 0.001
GGT (µkatal/l)	1745	0.43 (0.35; 0.56)	0.64 (0.48; 0.93)	< 0.001
HbA1c %	1745	5.1 (4.8; 5.4)	5.3 (5; 5.6)	< 0.001
**Glucose (mmol/l)**				
Fasting	1746	5.2 (4.9; 5.6)	5.7 (5.3; 6.1)	< 0.001
2-h	1622	5.6 (4.8; 6.6)	6.6 (5.6; 8.1)	< 0.001
**Insulin (µU/ml)**				
Fasting	1617	7.5 (5.5; 10.5)	13.8 (9.6; 20.1)	< 0.001
2-h	1619	39 (25; 58)	72 (47; 145)	< 0.001
HOMA-IR	1617	1.8 (1.2; 2.5)	3.5 (2.3; 5.3)	< 0.001
**OGTT**				< 0.001
NGT	918	710 (69%)	208 (35%)	
i-IFG	404	208 (20%)	196 (33%)	
i-IGT	87	39 (4%)	48 (8%)	
IFG + IGT	133	49 (5%)	84 (14%)	
Undiagnosed T2DM	80	17 (2%)	63 (10%)	

Data are given as absolute number and percentage for categorical data and as median (25th and 75th percentiles) for continuous data.

*ALT* alanine aminotransferase, *AST* aspartate aminotransferase, *GGT* γ-glutamyl transpeptidase, *HbA1c* Glycated Hemoglobin, *HOMA-IR* Homeostasis model assessment-insulin resistance index, *NGT* normal glucose tolerance, *i-IFG* isolated impaired fasting glucose, *i-IGT* isolated impaired glucose tolerance, *OGTT* oral glucose tolerance test, *T2DM* type 2 diabetes mellitus. To calculate p value chi-square tests were used for categorical variables and Wilcoxon rank-sum tests for continuous variables.

We observed that individuals having hepatic steatosis derived from MRI were older, comprised more males, had a higher BMI as well as higher levels of HbA1c, fasting glucose, 2-h glucose, fasting insulin, 2-h insulin, and HOMA-IR compared to those without hepatic steatosis. Individuals with hepatic steatosis through MRI had slightly higher levels of ALT, AST, and GGT compared to those without hepatic steatosis. Similarly, individuals with MRI-based definition of hepatic steatosis had more often prediabetes (i-IGT, i-IFG, IFG + IGT) or undiagnosed type 2 diabetes mellitus than individuals without hepatic steatosis (Table 1).

**Figure 1:**
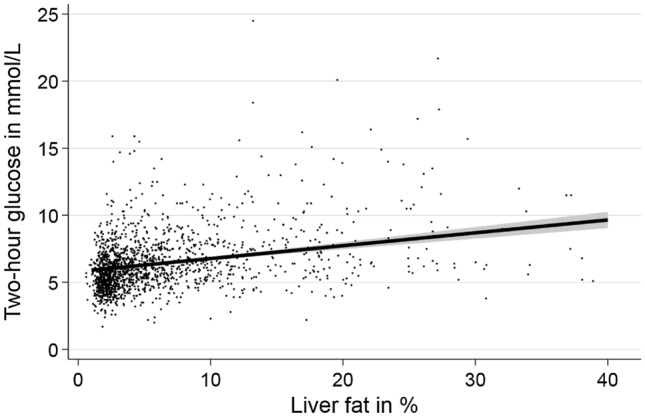
Association between liver fat fraction derived from quantitative MRI and two-hour glucose based on linear regression adjusted for age, sex, body mass index and alcohol consumption.

Linear regression models adjusted for age, sex, BMI and alcohol consumption revealed significantly positive associations between hepatic steatosis defined either by ultrasound or MRI. Levels of HbA1c, fasting glucose, 2-h glucose, fasting insulin, 2-h insulin and HOMA-IR were also associated with hepatic steatosis derived from ultrasound or MRI (Table 2). The mean level of 2-h glucose increased over the amount of fat in the liver (Fig. 1).

**Table 2:** Associations between hepatic steatosis derived from ultrasound and PDFF-MRI with continuous markers for glucose metabolism adjusted for age, sex, BMI and alcohol consumption.

Outcome variables	Hepatic steatosis (Ultrasound)		Hepatic steatosis (MRI)		MRI-PDFF %	
	β (95% CI)	P value	β (95% CI)	P value	β (95% CI)	P value
HbA1c %	0·07 (0·01; 0·12)	0.012	0·09 (0·03; 0·14)	0.004	0·01 (0·01; 0·01)	< 0.001
Fasting glucose (mmol/l)	0.18 (0.11; 0.24)	< 0.001	0.24 (0.17; 0.32)	< 0.001	0.03 (0·02; 0·03)	< 0.001
2-h glucose (mmol/l)	0.52 (0.31; 0.73)	< 0.001	0.75 (0.53; 0.97)	< 0.001	0.03 (0.02; 0.03)	< 0.001
Fasting insulin (µU/ml)	4.2 (3.2; 5.2)	< 0.001	4.9 (3.8; 5.9)	< 0.001	0.5 (0.4; 0.6)	< 0.001
2-h insulin (µU/ml)	22.3 (16.4; 28.2)	< 0.001	36.9 (30.8; 43.0)	< 0.001	3.5 (3.0; 4.0)	< 0.001
HOMA-IR	1.2 (0.9; 1.5)	< 0.001	1.4 (1.1; 1.8)	< 0.001	0.1 (0.1; 0.2)	< 0.001

β, derived from linear regression adjusted for age, sex, BMI and alcohol consumption; *95% CI* Adjusted 95% confidence interval, *BMI* body mass index, *HOMA-IR* homeostasis model assessment-insulin resistance index, *PDFF* proton density fat fraction.

**Figure 2:**
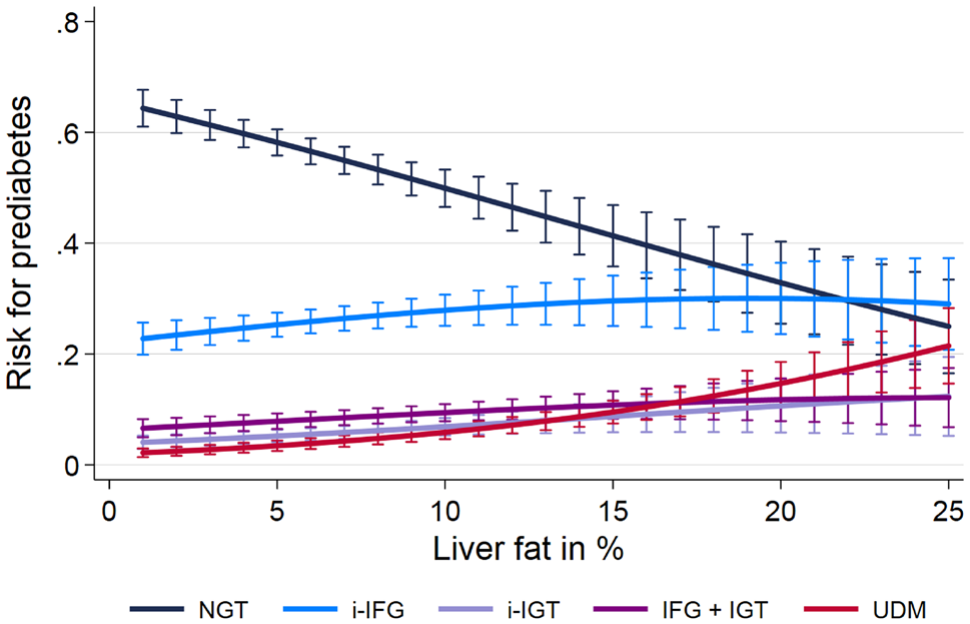
Association between liver fat and prediabetes and expressed as absolute risks based on multinomial regression after adjustment for age, sex, body mass index and alcohol consumption. *NGT* normal glucose tolerance test, *i-IFG* isolated impaired fasting glucose, *i-IGT* isolated impaired glucose tolerance, *UDM* undiagnosed type 2 diabetes.

Table 3 shows the associations between hepatic steatosis and prediabetes and undiagnosed type 2 diabetes mellitus using multinomial logistic regression models adjusted for age, sex, BMI and alcohol consumption. Individuals with hepatic steatosis defined either by ultrasound or MRI had a higher relative risk ratio to be in one of the prediabetes groups (i-IFG, i-IGT, IFG + IGT) or to have undiagnosed type 2 diabetes mellitus than individuals without hepatic steatosis. All associations were stronger when hepatic steatosis was defined by MRI compared to the definition from ultrasound. We observed a positive continuous association between the liver fat as assessed by MRI with prediabetes (Fig. 2).

**Table 3:** Associations of hepatic steatosis with categories of prediabetes and undiagnosed type 2 diabetes mellitus.

Outcome variables	Hepatic steatosis (Ultrasound)		Hepatic steatosis (MRI)		MRI-PDFF %	
	RRR 95% CI	P value	RRR 95% CI	P value	RRR 95% CI	P value
i-IFG	1.5 (1.2; 2.0)	0.002	1.6 (1.2; 2.2)	0.001	1.1 (1.0; 1.1)	< 0.001
i-IGT	1.7 (1.1; 2.8)	0.029	3.3 (2.0; 5.6)	< 0.001	1.1 (1.0; 1.1)	< 0.001
IFG + IGT	2.1 (1.4; 3.3)	< 0.001	2.5 (1.6; 3.9)	< 0.001	1.1 (1.0; 1.1)	< 0.001
Undiagnosed T2DM	2.8 (1.6; 4.8)	< 0.001	4.8 (2.6; 9.0)	< 0.001	1.2 (1.1; 1.2)	< 0.001

Multinomial regression with normal glucose tolerance (NGT) as base outcome adjusted for age, sex, BMI and alcohol consumption. *RRR* relative risk ratio, 95% confidence interval (CI), adjusted 95% confidence interval; *BMI* body mass index, *i-IFG* isolated impaired fasting glucose, *i-IGT* isolated impaired glucose tolerance, *T2DM* type 2 diabetes mellitus, *PDFF* proton density fat fraction.

To investigate a potential effect modification by sex on our associations we tested the interaction term of hepatic steatosis defined by MRI or ultrasound with sex on all outcomes. In none of these analyses, we observed any significant interactions.

## Discussion

In the present study, we investigated the association of hepatic steatosis derived from transabdominal ultrasound and MRI with prediabetes and undiagnosed type 2 diabetes mellitus in the general adult population. We demonstrated positive associations of hepatic steatosis with markers of glucose metabolism including HbA1c, fasting glucose, 2-h glucose, fasting insulin, 2-h insulin, and HOMA-IR. Similarly, we observed that individuals with hepatic steatosis had a higher risk of prediabetes or undiagnosed type 2 diabetes mellitus than individuals without hepatic steatosis. Associations were consistently stronger for hepatic steatosis derived from MRI compared to the ultrasound-based assessment.

Previous literature demonstrated associations between hepatic steatosis and type 2 diabetes mellitus^9,12–17^, but only few studies investigated the association between sonographically assessed hepatic steatosis and prediabetes in general populations^16,20–23^. A large occupational cohort of Chinese men showed that hepatic steatosis was a risk factor for prediabetes ascertained by OGTT after a follow-up of 5 years^20^. Similar results were observed in a longitudinal study with Japanese health-checkup participants defining IFG by fasting glucose levels^22^. Another study with a relatively small sample size (n = 213) demonstrated an association between hepatic steatosis and incident prediabetes defined by fasting glucose or HbA1c after a follow-up of 7 years^23^.

In line with our finding, data from the cross-sectional German KORA F4 study showed that subjects having hepatic steatosis as derived from fatty liver index (as calculated from BMI, waist circumference, GGT and triglycerides)^35^ had an increased chance to be in one of the prediabetes groups as defined by the ADA criteria^24^. In contrast to our results, a cohort study in 508 healthy subjects with a follow-up of five years failed to demonstrate a significant association of hepatic steatosis with incident prediabetes as defined by OGTT^16^. The discrepant finding may be explained by differences in study design and over-adjustment in the previous study^16^. For example, smoking or blood pressure are not considered as co-variables for the investigated association, because they do not confound the association between hepatic steatosis and metabolic endpoints.

Also a cross-sectional study from India^21^ did not find any association of hepatic steatosis with prediabetes categories as defined by the ADA criteria. Although that study adjusted for similar confounders (age, gender and waist circumference) as we did, probably no association was found due to the relatively small sample size of (n = 541) participants in that study^21^.

In our study we assessed hepatic steatosis by both ultrasound and MRI. We observed that the effect sizes for the association of hepatic steatosis with markers of prediabetes and undiagnosed type 2 diabetes mellitus were consistently larger when defining hepatic steatosis by MRI. This can be explained by the fact that MRI is a more sensitive and specific than ultrasound to detect liver fat^25^. Similarly, compared to ultrasound MRI is operator independent and has a lower sample variability^36^. MRI is highly reproducible and need less time for the examination of the entire liver^25^. Further, liver fat assessment by MRI is less confounded by body fat than liver fat measurement by ultrasound^37^.

It has been proposed that excessive lipid metabolites like diacylglycerol and ceramides within the liver cause insulin resistance by reducing phosphorylation of insulin receptor substrate 1 and 2 and activation of proinflammatory receptors^38^. An experimental study in mice suggested that diacylglycerol promotes insulin resistance in liver steatosis^39^. As a consequence, insulin is unable to suppress intrahepatic gluconeogenesis and lipolysis in adipose tissue, while promoting de novo hepatic lipogenesis^40^. In hepatic steatosis, endoplasmic reticulum stress and mitochondrial dysfunction may induce oxidative stress, which leads to production of reactive oxygen species^41^. As a result, β-cells of the pancreas are unable to compensate for the oxidative stress, which may lead to type 2 diabetes mellitus^42,43^. Recently, it has been investigated that various types of hepatokines such as fetuin A and B secreted by hepatocytes are increased in hepatic steatosis resulting in decreased insulin signaling, inflammation, lipolysis and insulin resistance^44^.

The association of hepatic steatosis with prediabetes and undiagnosed type 2 diabetes mellitus may be bidirectional as suggested from studies in patients with type 2 diabetes mellitus^8–10^. Similarly, there are hereditary factors to cause hepatic steatosis, which is then accompanied by insulin resistance and type 2 diabetes mellitus suggesting that liver fat may be a consequence rather than a cause of insulin resistance and type 2 diabetes mellitus^45^.

One strength of our study is the large population-based sample. Further, we defined hepatic steatosis according to sophisticated MRI analysis, which is more sensitive and specific than ultrasound^46^, because the threshold for detecting fat is lower and liver fat can be differentiated from liver iron^26^. Prediabetes was derived from the ADA criteria. Besides OGTT, we included further markers of glucose metabolism including HbA1c, fasting insulin, 2-h insulin and HOMA-IR.

A limitation of our study is that associations were only investigated cross-sectionally. Thus, we cannot draw causal inference. However, previous genomic studies using mendelian randomization demonstrated a causal relationship between hepatic steatosis and type 2 diabetes mellitus^47,48^. Although we adjusted our analysis for confounding, we cannot exclude residual confounding. Similarly, due to ethical constrains in our population of volunteers we did not use biopsy, which is the gold standard method to determine hepatic steatosis, or computed tomography as a radiation-based examination method^49^.

## Conclusions

Hepatic steatosis is associated with prediabetes and undiagnosed type 2 diabetes mellitus in the general population. The PDFF derived from liver MRI seems to be the more sensitive and specific method to determine hepatic steatosis than ultrasound, because it revealed stronger associations between hepatic steatosis and prediabetes.
